# Conserved regions of ribonucleoprotein ribonuclease MRP are involved in interactions with its substrate

**DOI:** 10.1093/nar/gkt432

**Published:** 2013-05-21

**Authors:** Olga Esakova, Anna Perederina, Igor Berezin, Andrey S. Krasilnikov

**Affiliations:** Department of Biochemistry and Molecular Biology and Center for RNA Molecular Biology, Pennsylvania State University, University Park, PA 16802, USA

## Abstract

Ribonuclease (RNase) MRP is a ubiquitous and essential site-specific eukaryotic endoribonuclease involved in the metabolism of a wide range of RNA molecules. RNase MRP is a ribonucleoprotein with a large catalytic RNA moiety that is closely related to the RNA component of RNase P, and multiple proteins, most of which are shared with RNase P. Here, we report the results of an ultraviolet-cross-linking analysis of interactions between a photoreactive RNase MRP substrate and the *Saccharomyces cerevisiae* RNase MRP holoenzyme. The results show that the substrate interacts with phylogenetically conserved RNA elements universally found in all enzymes of the RNase P/MRP family, as well as with a phylogenetically conserved RNA region that is unique to RNase MRP, and demonstrate that four RNase MRP protein components, all shared with RNase P, interact with the substrate. Implications for the structural organization of RNase MRP and the roles of its components are discussed.

## INTRODUCTION

RNase MRP is a catalytic ribonucleoprotein complex that is closely related to RNase P [a universal RNA-based enzyme responsible for the maturation of the 5′-end of tRNA and involved in some other activities (1)]. RNase MRP is an essential eukaryotic site-specific endoribonuclease (2,3) with a specificity distinct from that of RNase P. RNase MRP has been identified in practically all eukaryotes analyzed (4,5). RNase MRP is found primarily in the nucleolus (2,6–8) and—transiently—in the cytosol (9). A relatively low quantity of RNase MRP can also be found in the mitochondria, but mitochondrial RNase MRP has a distinct protein composition and specificity (10) and will not be discussed in this work.

RNase MRP is involved in the maturation of the 5.8S rRNA, cleaving the precursor molecule at a specific site (A3) within the internal transcribed spacer 1 (11–14). RNase MRP may participate in additional, earlier steps of rRNA maturation (15), but the exact nature of this activity has not been determined. RNase MRP was shown to be involved in the regulation of the cell cycle in yeast by participating in the cleavage of specific mRNAs (16–18), in the processing of U2 snRNA, as well as in the metabolism of a number of other RNAs (18–20). Defects in the activity of RNase MRP result in a variety of pleiotropic diseases in humans (21–23).

**Figure 1.**
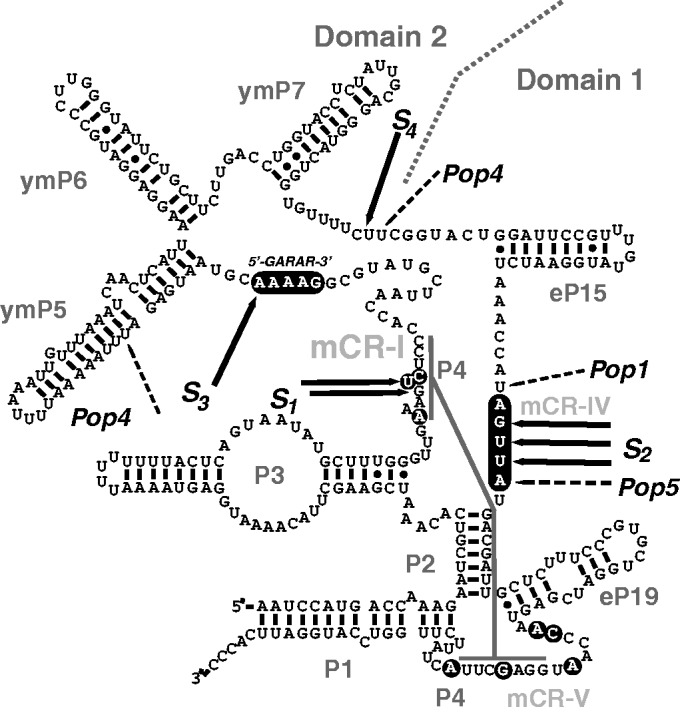
Secondary structure of the *S. cerevisiae* RNase MRP RNA (NME1). Phylogenetically conserved nucleotides (33), including the 5′-GARAR-3′ element (4) (where ‘R’ designates purines), are highlighted in black. Substrate cross-linking sites are shown by solid arrows and marked by ***S_1_***, ***S_2,_ S_3,_ S_4._*** Cross-linking sites for protein components Pop1, Pop4 and Pop5 [as determined in (34,35)] are shown by dashed lines. The secondary structure and the nomenclature of elements are based on previously published data (36–38).

*Saccharomyces cerevisiae* RNase MRP [reviewed in (24–26)] contains a 340-nt-long RNA component (NME1) and 10 essential proteins, eight of which (Pop1, Pop3, Pop4, Pop5, Pop6, Pop7, Pop8 and Rpp1) are shared with RNase P (27), and two [Snm1 (28) and Rmp1 (29)] that are unique to RNase MRP. Human RNase MRP has a similar composition (5,30–33). The RNA component of RNase MRP contains a domain (Domain 1 in Figure 1) that closely resembles the catalytic (C-) domain of RNase P, sharing the major secondary structure elements and several of the conserved nucleotides that are universally found in RNase Ps throughout the three domains of life (4,33,36,39) [reviewed in (26)]; the shared elements are involved in the formation of the catalytic core in bacterial RNase P (40–42) and, by inference, in eukaryotic RNase P and RNase MRP. The structural organizations of the C-domain in eukaryotic RNase P and Domain 1 in RNase MRP seem to be similar, and the two RNA domains interact with the same set (or similar sets) of proteins that are common to RNases P and MRP, including (but possibly not limited to) proteins Pop1, Pop5, Pop6, Pop7, Pop8 and Rpp1 (30,34,35,37,43–47).

Domain 2 of the RNA component of RNase MRP (Figure 1) and its RNase P counterpart—the specificity (S-) domain—do not have apparent sequence similarities [reviewed in (26)]. However, at least two proteins that are shared by RNase P and RNase MRP (Pop1 and Pop4) interact with both the S-domain of RNase P and the Domain 2 of RNase MRP (35,47), indicating a degree of (perhaps local) structural similarity between these two diverse domains. The S-domain of RNase P has a phylogenetically conserved part (39) that is involved in the recognition of the T- and D-loops of pre-tRNA substrates (40,42,48), but this part is missing in RNase MRP, consistent with distinct substrate specificities of the two enzymes. Overall, Domain 2 does not seem to be highly conserved when RNases MRP from different organisms are compared (33).

The only Domain 2 region that demonstrates a high degree of phylogenetic conservation is a 5′-GARAR-3′ (sometimes truncated to 5′-GARA-3′, where R is a purine) element (4,33) (Figure 1). This element was suggested to reside on top of a short helical stem (‘P8’) (4,33); however, this stem was not observed in the *S. cerevisiae* RNase MRP holoenzyme (37).

*In vitro* selection experiments have identified a weak sequence consensus for single-stranded RNase MRP substrates with a U-rich region 5′ to the cleavage site (positions −7 through −3) and an absolutely conserved cytosine at the position +4 (49); this pattern of RNase MRP cleavage is distinct from the pattern observed for the cleavage of single-stranded RNAs by RNase P (50).

The elements of RNase MRP RNA and protein components that are involved in interactions with RNase MRP substrates were not known. Here, we present the results of a cross-linking analysis of interactions of the RNase MRP holoenzyme with its substrate, and we specify the regions of RNase MRP RNA that are interacting with the substrate, as well as the identities of the involved RNase MRP proteins.

## MATERIALS AND METHODS

### *Saccharomyces cerevisiae* strains

*Saccharomyces cerevisiae* strains Pop3-BTtag, Pop4-BTtag, Pop5-BTtag, Pop6-BTtag, Pop7-BTtag, Pop8-BTtag, Rpp1-BTtag and Snm1-BTtag were based on yeast strain OE1004 (*MAT***a** RMP1::TAPHIS8::TRP1 *sep 1::URA3 pep4::LEU2 nuc1::LEU2 ade2-1 trp1-1 his3-11,15 can1-100 ura3-1 leu2-3,112*) (49). In addition to the affinity purification tag attached to the carboxyl terminus of RNase MRP protein component Rmp1 (49), each strain contained a 7.7 kDa peptide fused to the C-terminus of one of the RNase MRP protein components (Pop3, Pop4, Pop5, Pop6, Pop7, Pop8, Rpp1 and Snm1, respectively). The peptide, a 75 amino acids segment of *Propionibacterium shermanii* transcarboxylase (AGKAGEGEIPAPLAGTVSKILVKEGDTVKAGQTVLVLEAMKMETEINAPTDGKVEKVLVKERDAVQGGQGLIKIG), was known to become efficiently biotinylated in yeast (51) and served to noticeably decrease electrophoretic mobilities of the modified RNase MRP proteins. The peptide was fused to the proteins of interest using standard polymerase chain reaction-based methods with *HIS3* as the selectable marker. The presence of the peptide did not affect yeast viability.

*Saccharomyces cerevisiae* strain Rmp1-TAPtag was generated by the fusion of a tandem affinity purification (TAP) tag (52) to the C-terminus of the RNase MRP protein Rmp1, using strain LSY389-34A (*MAT***a** *sep 1::URA3 pep4::LEU2 nuc1::LEU2 ade2-1 trp1-1 his3-11,15 can1-100 ura3-1 leu2-3,112*) (29) (a generous gift from Mark Schmitt) as the starting point. The TAP-tag was fused using standard polymerase chain reaction-based methods with *TRP1* as the selectable marker. The addition of the tag did not affect yeast viability.

All yeast constructs were verified by sequencing of the affected regions.

### Isolation of RNase MRP holoenzymes

Active RNase MRP holoenzymes were isolated from yeast strains OE1004 (49), Pop3-BTtag, Pop4-BTtag, Pop5-BTtag, Pop6-BTtag, Pop7-BTtag, Pop8-BTtag, Rpp1-BTtag and Snm1-BTtag (as aforementioned) using a tandem affinity tag fused to the carboxyl terminus of RNase MRP protein component Rmp1 as previously described (49). The affinity tag was based on the common TAP-tag (52), but the original calmodulin-binding fragment was replaced with a His_8_ tag to maintain the presence of magnesium during RNase MRP isolation; RNase MRP purification was performed as follows (49).

Approximately 150 g of yeast paste was washed with water, resuspended in a buffer containing 20 mM Tris–HCl, pH 7.9, 150 mM KCl, 1 mM Mg–acetate, 10% glycerol, 1 mM phenylmethanesulfonylfluoride (PMSF), 0.1 mM ethylenediaminetetraacetic acid (EDTA) and disrupted using a BeadBeater (Biospec). Tween 20 was added to 0.1% (v/v), and the extract was clarified by centrifugation at 17 000*g* for 10 min (4°C) followed by 100 000*g* for 3 h (4°C). Three milliliters of rabbit IgG agarose (Sigma) was added to the clarified extract. After 5 h of incubation (4°C, with light agitation), the IgG agarose was washed six times with five volumes of the buffer containing 20 mM Tris–HCl, pH 7.9, 150 mM KCl, 1 mM Mg–acetate, 10% glycerol, 1 mM PMSF, 0.1 mM EDTA, 0.1% (v/v) Tween 20 (Buffer A) and resuspended in 2 ml of the same buffer. Three hundred units of tobacco etch virus (TEV) protease were added, and the sample was incubated overnight at 4°C with light agitation. The supernatant was collected, and the resin was additionally washed twice with 5 ml of buffer A. The supernatant fractions were combined; the buffer was exchanged for buffer B [20 mM Na–4-(2-hydroxyethyl)-1-piperazineethanesulfonic acid (HEPES), pH 7.9, 150 mM KCl, 1 mM Mg–acetate, 10% glycerol, 1 mM PMSF, 0.1 mM EDTA and 0.1% (v/v) Tween 20] and concentrated to the final volume of 2 ml using an Amicon-Ultra 15 (100 kDa MWCO) concentrator (Millipore). Following that, 0.5 ml of Ni-NTA agarose (Qiagen) was added to the sample. After 5 h of incubation (4°C, with light agitation), the resin was washed six times with 10 ml of buffer A supplemented with 10 mM Na–imidazole (pH 7.4). Following the final wash, RNase MRP was eluted using 10 ml of a buffer containing 400 mM Na–imidazole, pH 7.4, 50 mM KCl, 1 mM Mg–acetate, 10% glycerol, 1 mM PMSF and 0.1% (v/v) Tween 20 (20 min at 4°C with light agitation). The elution buffer was exchanged for a buffer containing 20 mM Tris–HCl, pH 8.0, 150 mM KCl, 1 mM Mg–acetate, 5 mM dithiothreitol (DTT) and 0.1% (v/v) Tween 20 (buffer C), and the sample was concentrated using an Amicon-Ultra 4 (100 kDa MWCO) concentrator (Millipore). The enzyme was transferred into buffer C supplemented with 50% (v/v) glycerol and stored at −20°C.

RNase MRP isolation from yeast strain Rmp1-TAPtag (as aforementioned) was performed following a previously described TAP-based protocol (37), but the step involving affinity purification on a calmodulin resin was skipped, and the sample was concentrated and used directly after tobacco etch virus protease cleavage.

### Synthesis of photoreactive RNase MRP substrate

The photoreactive (4-thiouridine-containing) RNase MRP substrate was synthesized using run-off transcription with T7 RNA polymerase and a synthetic deoxyribooligonucleotide as the template (53). Synthesis was performed at 37°C for 5 h in a buffer containing 40 mM Tris–HCl (pH 8.1), 6 mM MgCl_2_, adenosine triphosphate, cytidine triphosphate, guanosine triphosphate (GTP) (1 mM each), 1 mM 4-thioUTP (TriLink Biotechnologies), 5 mM DTT, 1 mM spermidine, 50 μg/ml bovine serum albumin, 100 nM DNA template oligonucleotide (5′-TTTGTGTTAAAAAATTTGTTGCC TATAGTGAGTCGTATTA-3′), 300 nM of oligonucleotide 5′-TAATACGACTCACTATAGG-3′, 0.2 U/μl SUPERase^.^In RNase inhibitor (Ambion) and 0.1 mg/ml T7 RNA polymerase. The resulting 4-thiouridine-containing RNase MRP substrate [5′-GGCAACAAAUUUUUUA*ACACAAA-3′ (where the RNase MRP cleavage site is indicated by the asterisk, (49) and 4-thiouridines are underlined] was purified using 10% denaturing (8 M urea) polyacrylamide gels and stored at −70°C.

When a radiolabeled substrate was required, the substrate was dephosphorylated with alkaline phosphatase and 5′-end ^32^P-labeled with T4 polynucleotide kinase. After labeling, the substrate was gel purified again and stored at −70°C. Alternatively, internally ^32^P-labeled substrate 5′-GGCAACAAAUUUUUUA*ACACAGAGAAA-3′ (RNase MRP cleavage sites are indicated by asterisks, and 4-thiouridines are underlined) was synthesized in the presence of ^32^P-α-GTP. The cross-links detected using the 5′- and internally ^32^P-labeled substrates were identical.

Exposure of 4-thiouridine and 4-thiouridine-containing RNA to light was avoided at all steps.

### Cross-linking experiments

Cross-linking of the photoreactive (4-thiouridine-containing) RNase MRP substrate to the isolated RNase MRP holoenzyme was performed in 96-well microtiter plates on ice. The RNase MRP holoenzyme (1.5 pmol) and the photoreactive substrate (1.5 or 15 pmol) were mixed in 10 μl of a buffer containing 10 mM Na–HEPES (pH 7.5), 50 mM NaCl, 10 mM MgCl_2_ and exposed to ultraviolet (UV) light (365 nm) generated by an 8 W handheld UV lamp kept at a 1-cm distance from the plate; the exposure time was 30 min.

### Detection of cross-linking sites on RNase MRP RNA

After UV cross-linking, DTT (to 5 mM) was added to the samples, the samples were incubated with 2 mg/ml of proteinase K for 30 min at room temperature, and then RNA was extracted with phenol followed by ethanol precipitation. The locations of the cross-links were identified using reverse transcription as previously described (37). Primers RTP1AL (complementary to nucleotides 324–340), RTP1B (316–340), RTP15A (243–276), RTP25 (168–201) and RTP4 (91–126) were used. The whole length of RNase MRP RNA was analyzed, except for 3′-terminal nucleotides 314–340.

### Identification of RNase MRP proteins that formed cross-links to the substrate

The photoreactive (4-thiouridine-containing) RNase MRP substrate was ^32^P-labeled and cross-linked to isolated RNase MRP holoenzymes as described earlier in the text. After cross-linking, 10 μl of a loading buffer [120 mM Tris–HCl (pH 6.8), 4% sodium dodecyl sulfate (SDS), 20% (v/v) glycerol, 2 M urea and 400 mM DTT] was added to the samples, the samples were incubated 5 min at 95°C and analyzed on 12% denaturing SDS–polyacrylamide gels. The radioactive bands were visualized using a PhosphorImager (Molecular Dynamics).

## RESULTS

### Interactions between the RNA component of the RNase MRP holoenzyme and its substrate

To identify regions of the RNA component of RNase MRP that were involved in interactions with substrates, we isolated the active RNase MRP holoenzyme from *S. cerevisiae*, subjected it to UV irradiation in the presence of a photoreactive RNase MRP substrate, and we located the sites of the UV-induced RNA–RNA cross-links that appeared in the presence of the photoreactive substrate (for details see ‘Materials and Methods’ section).

The design of the substrate used in cross-linking experiments was based on the results of *in vitro* selection of RNase MRP substrates (49), which demonstrated that a typical RNase MRP substrate has a CUC/CAC/CGC/UUC/AUC triad in the positions +2 to +4 relative to the cleavage site (with the cytosine at +4 being absolutely required for cleavage), no guanines in the positions +1 and −1 and (preferably) a U-rich stretch located 5′ to the cleavage site. The sequence of the substrate was 5′-GGCAACAAAUUUUUUA*ACACAAA-3′; the RNase MRP cleavage site is indicated by the asterisk.

**Figure 2.**
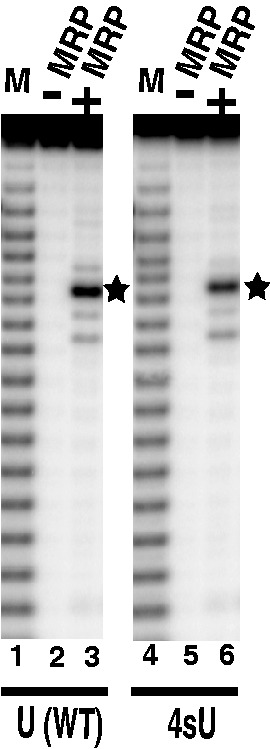
RNase MRP holoenzyme cleavage of the unmodified (lanes 1–3) and photoreactive (uridines substituted with 4-thiouridines, lanes 4–6) 5′-end ^32^P-labeled substrates (49). Lanes 1, 4: markers (alkaline hydrolysis); lanes 2, 5: substrates incubated in the cleavage buffer, but without RNase MRP; lanes 3, 6: RNase MRP cleavage. The cleavage site is shown by asterisks.

UV irradiation of the RNase MRP holoenzyme in the presence of the unmodified substrate did not result in the formation of detectable enzyme–substrate cross-links (data not shown). To increase the efficiency of the cross-linking to the levels that would make a reliable detection of transient enzyme–substrate complexes possible, we substituted uridines in our substrate with 4-thiouridines. The 4-thiouridine is structurally similar to uridine, the only difference being a single atom (oxygen for sulfur) substitution that has a minimal effect on the substrate’s properties but allows for a dramatically increased efficiency of cross-linking on photoactivation (54). The substitution of uridines with 4-thiouridines did not interfere with RNase MRP cleavage (Figure 2); all uridines were substituted with 4-thiouridines during *in vitro* transcription in the presence of 4-thiouridine and no uridine (‘Materials and Methods’ section).

**Figure 3.**
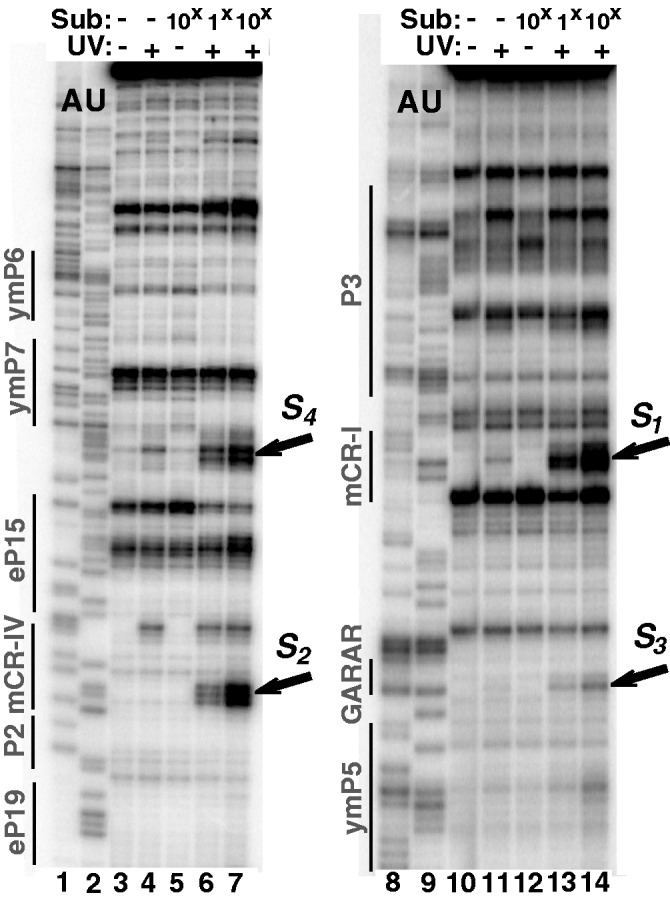
Cross-linking of a photoreactive substrate to the isolated RNase MRP holoenzyme: primer extension analysis. Lanes 1, 2, 8 and 9: sequence ladder; lanes 3 and 10: no substrate and no UV irradiation (control); lanes 4 and 11: UV irradiation in the absence of the substrate (control); lanes 5 and 12: substrate was present at a 10 to 1 substrate to enzyme molar ratio, but the sample was not UV-irradiated (control); lanes 6 and 13: UV cross-linking in the presence of the substrate (at a 1 to 1 substrate to enzyme molar ratio); lanes 7 and 14: UV cross-linking in the presence of the substrate (at a 10 to 1 substrate to enzyme molar ratio). The locations of secondary structure elements (Figure 1) are shown on the left. The locations of the identified substrate–RNase MRP RNA cross-links are indicated by arrows and marked ***S_1_*** to ***S_4_*** according to the labeling in Figure 1.

The modified substrate was exposed to UV light (365 nm) in the presence of RNase MRP (at a 1 to 1 and a 1 to 10 enzyme to substrate molar ratios), then the samples were deproteinated and analyzed by reverse transcription using primers specific to the RNA component of RNase MRP (‘Materials and Methods’ section). Typical results are shown in Figure 3; the locations of the identified cross-links are presented in Figure 1.

### Interactions between protein components of the RNase MRP holoenzyme and its substrate

**Figure 4.**
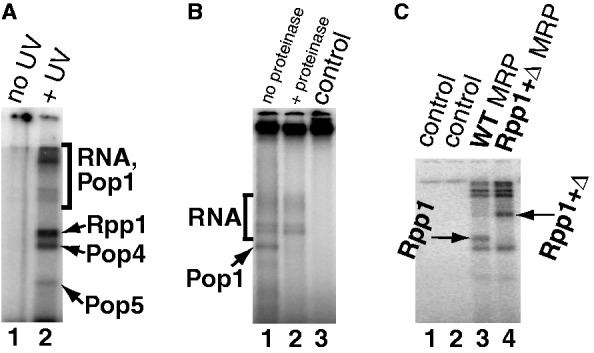
UV-induced cross-linking of a radiolabeled photoreactive RNase MRP substrate to the isolated RNase MRP holoenzyme. (**A**) Lane 1: control (no UV irradiation); lane 2: UV-induced cross-links. The identities of the RNase MRP components that are involved in the cross-links are shown on the right; see ‘Results’ section for explanation. Fifteen per cent denaturing (SDS) polyacrylamide gel. (**B**) Low-mobility radioactive bands resolved on a 6% denaturing (SDS) polyacrylamide gel. Lane 1: UV-induced cross-links; lane 2: same as lane 1, but the sample was treated with proteinase K to differentiate between protein and RNA bands; lane 3: control (no UV irradiation). (**C**) Identification of the band corresponding to the cross-link to the protein component Rpp1. Lane 3: UV-induced cross-links for the isolated ‘wild-type’ RNase MRP holoenzyme; lane 4: UV-induced cross-links for the isolated RNase MRP holoenzyme that had an elongated version of its protein component Rpp1 (denoted ‘Rpp1 + Δ’). The single band that had changed its mobility in lane 4 was identified as the one associated with Rpp1. Lanes 1 and 2: controls (same as lanes 3, 4, respectively, but without UV irradiation). Twelve per cent denaturing (SDS) polyacrylamide gel.

To identify protein components of RNase MRP that were involved in interactions with substrates, we used a ^32^P-radiolabeled version of the photoreactive substrate described earlier in the text. After UV-induced cross-linking between the RNase MRP holoenzyme and the radiolabeled substrate (taken at a 1 to 1 enzyme to substrate molar ratio), the samples were separated on denaturing SDS–polyacrylamide gels, and the radioactive bands were visualized (‘Materials and Methods’ section). Typical results are shown in Figure 4A.

The upper group of radioactive bands (Figure 4A) was identified as the product of cross-linking between the radiolabeled substrate (23-nt-long) and the largest protein component of RNase MRP, Pop1 (100 kDa), as well as products of cross-linking between the radiolabeled substrate and the RNase MRP RNA, NME1 (340-nt-long, 112 kDa). To differentiate between the substrate–proteins complexes of interest and the substrate–RNA complexes, we treated aliquots of the samples with proteinase K and used them as controls; the bands that were not sensitive to the proteinase K treatment were interpreted as substrate–RNA complexes (Figure 4B). The remaining (proteinase K-sensitive) band was the product of cross-linking of the substrate to Pop1, as confirmed by mass spectrometric analysis.

The identities of the higher-mobility products of cross-linking (Figure 4A) could not be reliably identified by mass spectrometry analysis because of the close electrophoretic mobilities of the remaining nine RNase MRP proteins [varying in sizes from 15.5 to 32.9 kDa (29)] and because of the shifts in mobilities caused by the formation of cross-linked complexes with the RNA substrate.

To help identify the remaining RNase MRP proteins that cross-linked to the substrate, we isolated RNase MRP from additional yeast strains that had small tags fused to each of the proteins: each strain had one specific ‘elongated’ RNase MRP protein (‘Materials and Methods’ section). The addition of a tag would decrease the electrophoretic mobility of the radioactive band associated with the protein that was tagged in a particular yeast strain but should not affect other bands. A typical result of such an experiment is shown in Figure 4C, where the elongated Rpp1 protein is denoted ‘Rpp1 + Δ’. We covered all RNase MRP proteins and were able to assign each of the remaining radioactive bands (Figure 4A). The results demonstrate that in addition to Pop1 (100.5 kDa), RNase MRP proteins Pop4 (32.9 kDa), Pop5 (19.6 kDa) and Rpp1 (32.2 kDa) cross-linked to the substrate.

## DISCUSSION

To better understand interactions of RNase MRP with its substrates, we performed cross-linking studies using a photoreactive (4-thiouridine-containing) substrate (49) and the RNase MRP holoenzyme isolated from yeast. The substitution of the uridines with 4-thiouridines in the substrate did not interfere with RNase MRP cleavage (Figure 2). The locations of the identified cross-links between the substrate and the RNase MRP RNA (NME1) are shown in Figure 1. Of the 10 RNase MRP protein components, four (Pop1, Pop4, Pop5 and Rpp1) were cross-linked to the substrate.

The identified cross-links between the substrate and the RNase MRP RNA are located in two distinct areas of the enzyme (Figure 1): in the Domain 2 near the junction with the Domain 1 (cross-links S_3_, S_4_), and in the phylogenetically conserved regions mCR-I, mCR-IV in the core of the Domain 1 (cross-links S_1_, S_2_). High-resolution structural information for the core of the Domain 1 is not yet available; however, low-resolution data (34,35,37,47), a high level of phylogenetic conservation (4,33,36,39), as well as the apparent close evolutionary relationship (4,5,55,56), all suggest that the structural organization of the core part of the Domain 1 (Figure 1) resembles that of the core of the catalytic (C-) domain (57) of bacterial RNase P [reviewed in (26)]. Several crystal structures of the C-domain of bacterial RNase P are available (40–42), and in the absence of the structure of the conserved core of the RNase MRP RNA Domain 1, the available structure of the core of the bacterial C-domain RNA can serve as its proxy.

**Figure 5.**
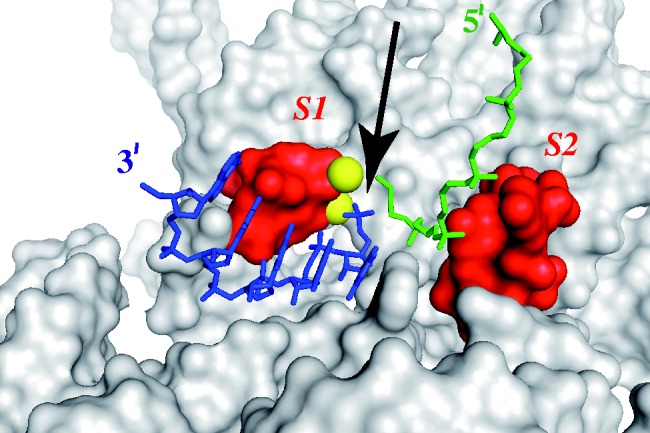
Using the crystal structure of bacterial RNase P in a complex with a product (42) as a proxy to position cross-links ***S_1_*** and ***S_2_***. The RNA component of the enzyme is shown in gray; the nucleotides corresponding to cross-links ***S_1_*** and ***S_2_*** are shown is red; the 5′ part of the cleaved substrate is shown in green; the 3′ part of the cleaved substrate in shown in blue. Magnesium ions are shown as yellow spheres. The location of the cleavage site is indicated by a black arrow.

Cross-link S_1_ (Figure 1) is located in a highly conserved region near the absolutely conserved bulged uridine found in all RNases MRP/P. In bacterial RNase P, this region coordinates two metal ions that are involved in catalysis [(42) and references therein]. Drawing parallels between RNase MRP and bacterial RNase P, this cross-linking site is within ∼5–8 Å of the expected position of the scissile bond in the substrate (42) (Figure 5). Further, in the case of yeast RNase P, single-stranded substrates were also found to form cross-links to this highly conserved area (50). Thus, the location of this cross-link is consistent with the expected overall similarity between the catalytic cores of the RNase MRP RNA and the ones in both eukaryotic and bacterial RNases P.

Cross-link S_2_ (Figure 1) is located in the area that is highly conserved in RNases MRP (4,33,36) and includes several nucleotides that are universally conserved in RNases P throughout the three domains of life (4,33,39) as well. In the crystal structure of the bacterial RNase P enzyme–product complex (42), the area corresponding to the S_2_ cross-link (Figure 5) is in the immediate vicinity (within ∼4–10 Å) of the phosphate backbone of the cleaved substrate 5′ to the cleavage site (the locations of the nucleobases were not identified in the crystal structure). Thus, the location of this (S_2_) cross-link could in principle serve as an indication that the RNase MRP substrate and the core of the Domain 1 are juxtaposed in a way resembling that of the core of the C-domain of bacterial RNase P and its substrate.

Cross-link S_3_ (Figure 1) is within the 5′-GARAR-3′ element, the only identified phylogenetically conserved element in the Domain 2 of RNase MRP (4,33). The structural organization of this region is not currently known. The presence of RNase MRP proteins result in significant conformational changes in this segment compared with the deproteinated RNase MRP RNA, but it is not clear if any proteins actually bind to the RNase MRP RNA there (35,37). The existence of this cross-link indicates that the 5′-GARAR-3′ element is positioned to interact with RNase MRP substrates, a role consistent with its phylogenetic conservation. It remains to be seen if this interaction involves any RNase MRP protein(s) as well.

Cross-link S_4_ (Figure 1) is located in the immediate vicinity of a previously identified binding site of the protein component Pop4 (35), suggesting that Pop4 is positioned to interact with the substrate. Indeed, Pop4 was one of the four RNase MRP proteins that were cross-linked to the photoreactive substrate (as aforementioned). It is not clear if Pop4 contributes to the specificity of the substrate recognition by RNase MRP or (being a basic protein) helps to bind the substrate in a non-specific fashion. It should be noted that in the related RNase P, Pop4 was shown to cross-link to poly(U) RNA, a potent inhibitor of the enzyme (50).

Pop5 and Rpp1 were also among the RNase MRP proteins cross-linked to the RNase MRP substrate. Pop5 and Rpp1 form a complex that binds to the RNase MRP Domain 1 RNA (34,35) (Figure 1) as well as to the C-domain of archaeal and eukaryotic RNases P (47,58–61). The archaeal/eukaryotic Pop5/Rpp1 complexes and the bacterial RNase P protein bind to matching parts of the respective RNA components and were proposed to play similar roles (34,60). Thus, given that the bacterial RNase P protein participates in interactions with the substrate [(42,62,63) and references therein], interactions of Pop5 and Rpp1 with the RNase MRP substrate are consistent with the overall similarity of the catalytic parts of RNases P/MRP throughout the three domains of life.

Pop1, the largest RNase MRP protein, is the fourth protein component interacting with the RNase MRP substrate. Although the role of Pop1 in RNase MRP is not yet known (64), the available low-resolution data indicate that it interacts with both of the RNase MRP RNA domains (35,47). The proximity of the substrate–RNase MRP RNA cross-link S_2_ to the previously identified (35) location of a Pop1 cross-link in the mCR-IV RNA region (Figure 1) can explain the Pop1-substrate cross-link; at the same time, given the large size of Pop1, it cannot be excluded that it has multiple contacts with the substrate. The large size of Pop1 and its involvement in interactions with both central and distal parts of RNases P/MRP (35,47), as well as the observed defects in RNase P/MRP assembly associated with Pop1 mutations (64), all suggested a role for Pop1 in stabilizing the overall structures of RNases P/MRP. Our cross-linking results show that Pop1 is positioned to play a role in substrate recognition as well.

Although RNase MRP is structurally and evolutionarily related to RNase P, the substrate specificities of the two enzymes differ (20,24–26,49,50,65–67). The recognition of the ‘canonical’ RNase P substrate, pre-tRNA, involves interactions between the T- and D-loops of the substrate and the specificity (S-) domain of the RNA component (40,42,48). Thus, given that pre-tRNA is not a substrate that is specifically recognized by RNase MRP, the loss of the S-domain in this enzyme seems logical. Does the RNase MRP Domain 2, which replaces the S-domain, play a role in RNase MRP substrate recognition? Our cross-linking results show that the phylogenetically conserved part of the Domain 2 (the 5′-GARAR-3′ element, Figure 1) interacts with the substrate; the determination of the exact role of this interaction in the substrate specificity of RNase MRP will require additional studies. RNase MRP cleavage absolutely requires the presence of cytosine four nucleotides 3′ to the cleavage site (49), whereas cleavage by RNase P does not (50). The conserved 5′-GARAR-3′ element, which is unique to RNase MRP and is found to interact with its substrate, could potentially be involved in interaction with the conserved cytosine of the substrate. It should be noted that, given the small size of the substrate used in this study, cross-links S_3_, S_4_ (Figure 1) must be located in a proximity to the catalytic core of the enzyme (cross-links S_1_, S_2_). Thus, it is likely that the parts of the RNase MRP RNA component that form cross-links S_3_, S_4_, fold back into the Domain 1, modifying the substrate-binding interface that otherwise would be similar to that in RNase P (given the similarity between the RNase P C-domain and Domain 2 in RNase MRP). It should be noted that RNase MRP cannot cleave double-stranded substrates (49), consistent with a more limited space near the catalytic site available for the substrate compared with that in RNase P.

Given the large size of the protein part of RNase MRP, it is expected that proteins play a significant role in determining the substrate specificity of the enzyme (66). It is somewhat surprising that proteins Snm1 (28) and Rmp1 (29), which differentiate the protein part of RNase MRP from that of RNase P, are not among the proteins found to cross-link to the RNase MRP substrate. Although it is possible that Snm1 and Rmp1 play roles other than RNase MRP substrate binding, one can speculate that these proteins play roles in interacting with (unidentified) protein parts of ribonucleoprotein substrates *in vivo*.

## FUNDING

National Institutes of Health (NIH) [GM085149]; American Heart Association [12GRNT10590001]. Funding for open access charge: NIH [GM085149].

*Conflict of interest statement.* None declared.
